# Neighborhood Violence Exposure and Alcohol and Tobacco Use Initiation Among Ethnic Minority Adolescents †

**DOI:** 10.3390/healthcare13020194

**Published:** 2025-01-19

**Authors:** Anna Maria Santiago, Iris Margetis

**Affiliations:** 1 School of Social Work, Michigan State University, East Lansing, MI 48824, USA; 2 Department of Economics, Michigan State University, East Lansing, MI 48824, USA; margetis@msu.edu

**Keywords:** alcohol use initiation, tobacco use initiation, exposure to neighborhood violence, natural experiments, Cox Proportional Hazards model, threshold effects

## Abstract

**Background/Objectives:** Although the extant literature has recognized the importance of neighborhood contexts for adolescent alcohol and tobacco use, less is known about the effects of exposure to neighborhood violence on the prevalence and timing of initiation across gender and race/ethnic groups. **Methods:** This secondary analysis of administrative and survey data from a natural experiment in Denver examines the influence of neighborhood contexts on the health and well-being of 1100 Latino/a and African American adolescents. Cox Proportional Hazard models were used to (1) estimate the effects of exposure to neighborhood violence on the prevalence and timing of adolescent alcohol and tobacco use initiation; (2) examine gender and race/ethnic variations in alcohol and tobacco use initiation after controlling for adolescent, caregiver, household, and other neighborhood characteristics; and (3) test for threshold effects. **Results:** Prevalence rates among all adolescents were 12.9% for alcohol use initiation and 13.7% for tobacco use initiation but were 14.6% and 17.3%, respectively, among adolescents exposed to higher levels of neighborhood violence. The average age of initiation was 16.1 and 15.6 years for alcohol and tobacco use, respectively, but 2–8 months earlier for adolescents exposed to higher levels of neighborhood violence. Heightened exposure to neighborhood violence increased the hazards of alcohol use initiation by 32% for all adolescents and 38% for adolescent males. The hazards of tobacco use initiation were 1.3 to 1.5 times higher for male, Latino/a, and African American adolescents. Exposure to neighborhood violence suggests threshold effects of diminishing returns on adolescent tobacco use initiation. **Conclusions:** Findings underscore the need to examine gender and race/ethnic group differences in adolescent alcohol and tobacco initiation, the multiple pathways to such use, and interventions aimed at reducing neighborhood violence.

## 1. Introduction

Although rates of alcohol and tobacco use among adolescents have continued their downward trajectory, both remain among the most commonly used and abused substances. Recent lifetime prevalence rates among U.S. adolescents were 21.6% for alcohol and 8.3% for tobacco use although these rates vary by ethnicity and gender [1,2]. Adolescent alcohol and tobacco use have been linked to adverse preventable adolescent and adult behavioral and physical health outcomes over the life course [3,4] including increased risky sexual behavior [5]; sexual victimization [6]; alcohol and tobacco dependence in adulthood [4,7,8,9,10]; some forms of cancers as well as premature morbidity and mortality [4,11,12,13]. Further, adolescents who use alcohol or tobacco have higher rates of delinquency [14]; weaker academic performance [15] and employment prospects [16] relative to their peers who abstain from such use. Thus, preventing or delaying initiation of both alcohol and tobacco use remain key public health goals.

Since the late 1980s, attention has shifted from a primary focus on individual, family, and peer risk factors [3,17,18,19,20,21,22,23] to examine the influence of the surrounding environment on adolescent substance use. Indeed, family, or individual behavioral problems as well as negative associations with peers are strongly influenced by environmental factors [3,14,19,20,24,25,26]. Prompted by the seminal work of Wilson [27], scholars have underscored the need to examine neighborhood- and community-level factors, such as social disorganization, limited informal social control, concentrated disadvantage or concentrated affluence, high levels of crime, and exposure to neighborhood violence, on adolescent health and risky behavioral outcomes including alcohol and tobacco use [23,26,28,29,30,31]. Prior work emphasizes the need to disentangle where, when, why, and for whom neighborhood contexts matter [32]. It also highlights the need to examine whether exposure to higher levels of neighborhood violence continues to increase the risk of alcohol and tobacco use initiation or if the relationship is one of diminishing returns once certain thresholds are reached [33,34,35].

In this study, we attempt to disentangle these effects and mechanisms to address the following research questions: (1) What are the effects of exposure to neighborhood violence on the prevalence and hazards of adolescent alcohol and tobacco use initiation? (2) Does the effect of neighborhood violence exposure on adolescent alcohol and tobacco use initiation vary significantly by gender and race/ethnicity after controlling for adolescent, caregiver, household, and other neighborhood contexts? and (3) Are there threshold effects between neighborhood violence exposure and adolescent alcohol and tobacco use initiation? We hypothesize that exposure to neighborhood violence (a) increases the hazards of alcohol and tobacco use initiation during adolescence; (b) decreases the age of alcohol and tobacco use initiation; (c) increases the hazards for adolescent male initiation of alcohol and tobacco use; (d) increases the hazards of alcohol and tobacco use initiation for Latino/a adolescents; and (e) exhibits a point of diminishing returns to both alcohol and tobacco use initiation.

To address these questions and hypotheses, we conducted a secondary data analysis using administrative and survey data originally gathered from a natural experiment in Denver for approximately 1100 Latino/a and African American adolescents aged 10 to 18 whose families were randomly assigned to public housing units and neighborhoods for an extended period of time during childhood. In this study, we employ the U.S. Office of Management and Budget definitions of Latino/a background and race. In the United States, persons of Latino/a background may be of Cuban, Mexican, Puerto Rican, Central or South American, or other Spanish culture or origin regardless of race (https://www.census.gov/topics/population/hispanic-origin/about.html, accessed on 30 September 2024). Likewise, the race category includes racial national origin or sociocultural groups. Thus, African American in the United States is a social definition of race that is based upon self-identification and reflects individuals having origins in any of the racial groups in Africa (https://www.census.gov/topics/population/race/about.html, accessed on 30 September 2024) (Only public housing residents who identified themselves and their children as Latino/a/Hispanic or African American/Black were included in this study since they were the focal populations of the study).

Our work advances the extant literature on exposure to neighborhood violence and adolescent alcohol and tobacco use initiation in four ways. First, because the primary caregivers of our study participants were quasi-randomly assigned to neighborhoods when they first entered public housing the challenge of parental geographic selection bias is addressed. Second, we utilize a composite measure of exposure to neighborhood violence that depicts such exposure at smaller spatial scales than the census tract while concurrently controlling for an array of adolescent, caregiver, household, and other neighborhood environmental contexts identified in the literature as influencing adolescent alcohol and tobacco use initiation. Third, in contrast to previous studies that have focused primarily on the experiences of White adolescents or by gender, we examine the differences in these effects for low-income Latino/a and African American adolescents and by varying levels of neighborhood violence exposure. Fourth, we assess the extent to which threshold effects are associated with the influence of exposure to neighborhood violence and adolescent alcohol and tobacco use initiation.

### 1.1. Gender, Racial, and Ethnic Variations in Adolescent Alcohol and Tobacco Use Initiation in the United States

Previous studies document considerable variation in the prevalence and timing of adolescent alcohol and tobacco use by gender and racial/ethnic background [36,37,38,39,40,41,42,43,44,45,46,47,48,49]. Although recent studies [10,36,37] suggest the gender gap in adolescent alcohol and tobacco use initiation is decreasing or disappearing, prevalence rates typically remain higher among males than females [2,38,39,40]. Also, multiple studies (e.g., [36,38,41,42,43,44,45,46]) report prevalence of adolescent alcohol and tobacco use is highest among White adolescents and lowest among African American adolescents with Latino/a adolescents typically falling in between or with rates similar to Whites. In particular, recent studies note tobacco smoking continues to decline among all adolescents [48] but is declining most rapidly among White adolescents and at a significantly slower pace among African American adolescents [37]. However, reasons for these differences remain poorly understood. Indeed, a growing number of scholars (e.g., [12,41,42,43,44,45]) have observed that individual, family, and peer factors were less significant predictors of substance use initiation among ethnic minority adolescents compared to White adolescents. Further research is needed to identify the mechanisms underlying these subgroup differences.

A lingering gap in the literature revolves around the factors associated with the timing of substance use initiation including recently noted increases in age of onset across most gender, racial, and ethnic groups [46,47,48,49]. Teens in the United States typically initiate their substance use during late childhood or early adolescence [13] with the average age currently around age 16 for both alcohol and tobacco use [46]. The average age of initiation was youngest for White teens and latest for African American teens with Latino/as falling in between the two groups [46,47,48,49]. Further, Bacio and colleagues [44] report variations in age of onset with native-born Latino/as more likely to initiate use at earlier ages than immigrant Latino/a adolescents. Nonetheless, the factors underlying variations in the timing of adolescent substance use initiation are less known partly because information about both the incidence and timing of substance use have not been always available in most data sources (see discussion in [8,10,12,26,50,51]).

### 1.2. How Neighborhoods Might Influence Adolescent Initiation of Alcohol and Tobacco Use

Ecological systems theory [52] argues that neighborhoods may influence adolescent behavior through an array of causal mechanisms operating through social, institutional, or biological processes [53,54,55,56,57,58,59,60,61,62,63,64,65,66,67,68,69,70,71]. A longstanding thread in this literature has linked social disorder and violence exposure to an array of problematic behaviors among adolescents [27,53,72] including alcohol and tobacco use initiation (see [73,74,75,76,77,78,79,80,81,82,83,84,85,86,87,88,89,90,91,92,93,94,95,96,97,98,99,100,101,102,103,104,105,106,107,108,109]). Exposure to neighborhood violence has been posited to trigger early initiation of alcohol and tobacco use as a way of coping with the heightened levels of stress associated with such exposure [31,77,78,79,82,83,86,90,92,94,96,97,98,100,109]. However, findings to date are mixed. On the one hand, exposure to heightened levels of neighborhood violence or perceived lack of safety in the neighborhood have been associated with increasing alcohol use [80,105], tobacco use [50,80,98,102,104], or multiple substances including alcohol and tobacco [91,92,103,105,106,107,108,109]. Yet, others have reported that exposure to neighborhood violence had no effect or reduced substance use initiation, specifically with alcohol use initiation [89,93,96,104,108]. Conversely, several studies [31,87,88,110] reported increased adolescent alcohol use in neighborhoods with higher perceived levels of neighborhood safety and affluence. Nonetheless, White and colleagues [103] argued the presence of social toxicity within communities, which includes violence, was a significant driver of substance abuse initiation among adolescents.

Recent work has examined the influence of proximity to neighborhood risks (see [60,81,84,105]). In several case studies, close proximity to and density of alcohol and tobacco outlets increased adolescent substance use [60,81,106]. Further, Haley and colleagues [106] reported increased alcohol use among adolescents residing in New York City neighborhoods that had both concentrated alcohol outlet density and higher levels of violent crime. However, in a national study conducted by Adachi-Mejia et al. [84], neither outlet density nor proximity to the adolescent’s place of residence was associated with adolescent smoking or smoking intensity.

Relatively few studies to date have assessed how exposure to neighborhood violence or social disorder influences gender, racial, or ethnic differences in adolescent alcohol or tobacco use initiation [41,60,67,74,77,94,102,108]. Heightened exposure to neighborhood violence was associated with higher levels of substance use, particularly smoking, for children [67,77,94] although those effects might be delayed for girls [77]. Some studies report increased alcohol and tobacco use by African American adolescents with exposure to greater neighborhood social disorder [41,67,74,102]. One of the consistent findings for Latino/a adolescents shows increased risks of tobacco use initiation with greater exposure to neighborhood violence [67,102]. Truong and colleagues [60] also report increased substance among Latino/a and African American adolescents with greater proximity to and density of alcohol outlets. In contrast, Zhao and colleagues [108] reported no significant racial or ethnic differences in the influence of neighborhood social disorder on adolescent substance use initiation.

With relatively few exceptions (see [67,71,83,102,111,112]), much of the aforementioned adolescent substance abuse literature is subject to numerous methodological challenges [58] with perhaps the most formidable one being geographic selection bias. Comprehensive reviews and critiques of the neighborhood effects literature on child and adolescent behaviors (see [55,58,59,69]) concluded that random-assignment experiments or natural quasi-experiments mimicking random assignment of households to neighborhoods are the strongest designs to estimate unbiased neighborhood effects and to generate reliable causal inferences. To our knowledge, only two studies using experimental or quasi-experimental assignment to neighborhood have been conducted to date that are relevant to understanding the causal effects of neighborhood on adolescent alcohol and tobacco use: the *Moving To Opportunity* (MTO] demonstration and the *Denver Child Study*.

In *Moving To Opportunity*, low-income African-American supportive housing residents were assigned to one of three groups: (1) control, (2) rental housing voucher, or (3) voucher that also included relocation counseling and a minimum one-year requirement that households move to low-poverty neighborhoods [83,111,112]. Follow-up assessments after assignment reported significant reductions in girls’ drinking but increased risk of boys’ smoking after several years of residence in less disadvantaged neighborhoods [83,111]. The *Denver Child Study* [67] examined quasi-random assignment to subsidized housing units and neighborhoods and its influence on outcomes over the course of childhood. Data from this study found an increased risk of tobacco use initiation for adolescent males and females as well as African American and Latino/a adolescents associated with increased levels of neighborhood crime. A significantly higher risk of alcohol use initiation was found for adolescent males living in high-crime neighborhoods. A recent paper by Lee and Santiago [102] examining cumulative exposure to neighborhood conditions during pre-adolescence on adolescent substance use found that exposure to neighborhood social disorder was associated with a 36% higher hazard rate of cigarette use but only for Latino/a adolescents. In the current study, we extend this prior work by examining gender and racial/ethnic differences in the prevalence and timing of alcohol and tobacco use and variations associated with the magnitude of exposure to neighborhood violence during adolescence measured at smaller neighborhood scales than traditional census tracts or zip code areas.

## 2. Materials and Methods

### 2.1. The Natural Experiment in Denver

In this study, we analyze deidentified administrative and survey data obtained from a unique natural experiment involving residents from the Denver (CO) Housing Authority’s (DHA) conventional and scattered site subsidized housing programs (for details see [67,113]). During the period between 1987 and 2010, eligible applicants on a unified waitlist were offered one of DHA’s vacant subsidized housing units suitable for their family size and the gender of their children. Unlike highly segregated public housing units in Midwestern cities like Chicago, Detroit, and Milwaukee, DHA’s 4500 subsidized housing units are located in approximately 60% of all neighborhoods in the congruent City and County of Denver. If eligible applicants did not accept the initial offer, they typically received a second offer in the same neighborhood for the next available unit suitable for their needs. Any applicant who did not accept either offer was dropped to the bottom of the waitlist extending their wait time for a year or more.

Nearly 90% of eligible applicants accepted their initial or second offers and 75% accepted units in their originally assigned neighborhoods. In response to those who did not accept their originally assigned neighborhoods and units, Santiago and colleagues conducted a series of statistical balancing tests to ascertain the extent to which DHA applicants were quasi-randomly assigned to their DHA neighborhoods and units (see description in [67,113]). Results show that initial assignment to a DHA housing unit closely simulated random assignment of households to neighborhood characteristics except for African American households. Findings suggest that they may not be randomly distributed across DHA developments or neighborhoods. Since Santiago and colleagues [67] could not determine whether these distributions were the product of any systematic actions by DHA staff members or because of geographic self-selection by African American applicants, all statistical analyses control for African American ethnicity to account for this inconsistency to random assignment.

### 2.2. Denver Child Study Retrospective Survey and Study Population

At the core of the *Denver Child Study* was a 90 min telephone or in-person retrospective survey administered to 550 current and former DHA residents identified as primary caregivers (in recognition that a nontrivial number of adolescents in the *Denver Child Study* were raised by grandparents or other family members), whose households and their 1149 children aged 10 to 18 entered DHA during the period of random assignment and who met minimum inclusion criteria for residence in DHA, having at least one child reside in the randomly assigned unit, and were of Latino/a or African American background [67]. These households completed interviews between 2006 and 2012. Since the outcomes of interest for this paper are alcohol and tobacco use during adolescence (which we define as the period between 10 and 18 years of age), the final study populations from the *Denver Child Study* include data for adolescents who: (1) were at least 10 years old at the time of the survey; (2) were randomly assigned to a DHA neighborhood prior to the initiation of the specific outcomes of interest; (3) resided in DHA for a minimum of two years; and (4) had complete information for all variables used in our analytical models. We lost 25 cases because of incomplete residential address information that prevented us from assigning an address to a census tract. Additional cases were lost because adolescents resided in DHA housing for less than two years (N = 24 in the alcohol use initiation analysis; N = 9 in the tobacco use initiation analysis). Finally, cases were lost if substance use initiation occurred before the age of 10 or prior to the move into the randomly assigned DHA housing unit and neighborhood (N = 38 from the alcohol use initiation analysis; N = 22 from the tobacco use initiation analysis). Since all study participants came from low-income public housing families, the small loss of cases did not introduce significant bias in the study populations. These criteria yielded final study populations of 1062 for alcohol use and 1093 for tobacco use.

### 2.3. Neighborhood Context Indicators

We used caregiver-identified residential locations to assign corresponding census tract codes which then allowed us to link these locations to a rich set of neighborhood indicators from the U.S. Census, the Piton *Neighborhood Facts* Database, and subjective indicators of neighborhood conditions derived from the *Denver Child Study*. Linear interpolation or extrapolation was used to derive annual estimates of neighborhood conditions for our entire study period [42]. Caregiver and adolescent qualitative responses about the influence of neighborhood conditions during adolescence also were examined to provide context regarding purported neighborhood mechanisms associated with adolescent alcohol or tobacco use initiation. Timing of outcome information was solicited from caregivers and used to temporally match outcomes with corresponding neighborhood indicators. In this study, neighborhood conditions were estimated contemporaneously with the initiation of alcohol or tobacco use. For adolescents who did not initiate either substance, indicators were measured at age at the time of the survey (for those under 18) or age 18 (for those over 18).

#### 2.3.1. Exposure to Neighborhood Violence

Our key predictor variable, exposure to neighborhood violence, reflects caregiver perceptions of neighborhood social problems which are measured at the more immediate local neighborhood level (e.g., surrounding block or block faces). Instead of using data from the U.S. *Uniform Crime Reports*, which are known to undercount the true volume of crime and are riddled with incomplete and inconsistent information across police departments [114], we opted to use a self-reported measure of exposure to violence that was developed for the *Denver Child Study.* In prior studies (e.g., [67]), Santiago and colleagues found that indicators depicting conditions in the immediate neighborhood were consistently more robust predictors than census-tract level indicators. Additionally, self-reports of exposure to violence or crime as victims or offenders often include incidents not reported to police or on official records [114,115]. The neighborhood social problems index (range 0–6) is incremented by a factor of “one” for each of the following conditions present in the neighborhoods where adolescents resided during childhood: people selling drugs; gang activity; homes broken into by burglars; people being robbed or mugged; people getting beaten or raped; children and youth getting into trouble. Children or youth getting into trouble is the closest measure available in the study to capture potential neighborhood peer effects. Effectively, this index captures some of the prominent property and violent crimes incorporated into the *Uniform Crime Reports* but at an immediate neighborhood scale instead of the larger census tract. The Cronbach’s alpha for the index was 0.831. As shown in Table 1, adolescents in our study lived in neighborhoods where the average social problems index score was approximately 1.9 for both the alcohol and tobacco initiation study populations.

#### 2.3.2. Other Neighborhood Covariates

We control for five other neighborhood characteristics in our models that the literature has identified as associated with alcohol and tobacco use initiation [55,67,99,101,102]. Census indicators for neighborhood ethnic composition (percent Latino/a and African American residents) and socioeconomic status (social vulnerability index and occupational prestige) typically have been identified as protective factors while vintage of housing stock (percent of housing units built before 1940) indicating older, housing distressed neighborhoods that may offer more opportunities for unsurveilled activities was identified as a potential risk factor. All were derived using the Geolytics *Neighborhood Change Data Base*. Based upon the results of principal components analysis across the multiple census years covered in the study, Santiago and colleagues [67] generated a highly reliable (α = 0.910) composite indicator of neighborhood disadvantage that incorporated neighborhood poverty rate, unemployment rate, percent renters, and the percentage of female-headed households. Their social vulnerability index ranged from a low of 0 indicating high levels of neighborhood advantage and 400 indicating high levels of neighborhood disadvantage. Using the *1989 General Social Survey* prestige scores calculated for each occupational type in the United States, we estimated neighborhood occupational prestige scores that were then weighted by the observed proportional distribution of occupations among those residing in the census tract neighborhood [116]. When all employees in the neighborhood are laborers, the prestige score is 29.44. Conversely, when all employees in the neighborhood are in professional occupations, the prestige score is 62.24.

Table 1 depicts the characteristics of neighborhoods where ethnic minority adolescents in our study resided as well as provides comparable neighborhood data for all neighborhoods in the City and County of Denver. Adolescents in our study resided in neighborhoods that were primarily occupied by other ethnic minority residents—55% Latino/a and roughly 15% African American. Neighborhood residents tended to work in laborer jobs possessing low levels of occupational prestige (mean = 37.4). Further, adolescents in the study were residing in neighborhoods with moderate levels of disadvantage (mean = 119). Denver adolescents as a whole resided in neighborhoods that were comprised of approximately 40% ethnic minority residents (29% Latino/a and 11.5% African American). Neighborhood residents had slightly higher levels of occupational prestige (mean = 41). These neighborhoods also were slightly more advantaged with an average social vulnerability score that was approximately 22 points lower than the neighborhoods where study participants resided. Of interest, both the study participants and Denver adolescents as a whole resided in neighborhoods where approximately one-quarter of the housing stock was built before 1940, suggesting these were older and potentially more distressed locations within the city.

#### 2.3.3. Adolescent, Caregiver and Household Covariates

Our analytical models also control for an array of adolescent, caregiver, and household characteristics that have been associated with the initiation of alcohol and tobacco use during adolescence (see Supplementary Table S1). In addition to controlling for the adolescent’s gender, race/ethnicity through the use of stratification, birth order serves as a proxy for potential sibling effects [117]. Previous studies (e.g., [19,23,44,85,99]) have incorporated caregiver characteristics including age, immigrant status, educational attainment, and earnings with all time-varying characteristics measured at the time of initiation of the specified behavior or age 18 if an adolescent did not initiate alcohol or tobacco use. Given the links between adolescent substance use initiation and maternal depression [118], we also identified the presence of depressive symptomology in caregivers using the Center for Epidemiological Studies-Depressive Symptomology (CES-D) inventory. Scores of 16 or higher on the CES-D as measured at the time of the survey indicated subclinical or clinical-level symptoms of depression. Household indicators include measures controlling for the number of siblings in the household [117]. As noted in recent studies [51,99,101], residential stability or lack thereof also plays an important role in increasing the risk of substance use initiation. We employ the number of residential moves from birth to the time of initiation of alcohol or tobacco use or age at the time of survey or age 18 if the adolescent did not use substances to capture such residential instability. Consistent with the work of Wodtke and colleagues [119], we utilized an index developed by Santiago and colleagues [67] to capture the level of household stress as a potential contributor to adolescents’ alcohol and tobacco use initiation. Five items measured the extent to which adolescents’ households experienced financial difficulties, unstable employment, illnesses or injuries, utility shutoffs, or housing evictions each year during adolescence. Higher index scores indicated higher levels of household stress.

What we did not include in our models were indicators for caregiver or peer substance use. Earlier models described in Santiago et al. (2014) [67] included caregiver substance abuse indicators but these were not found to be statistically significant, in part because we could not identify with any degree of precision the timing of caregiver alcohol and tobacco use relative to adolescent initiation of use. Additionally, our indicator for neighborhood social problems includes an item related to peer effects (children or youth getting into trouble). In previous analyses, we had parsed out the peer effect item from the other items in the neighborhood social problems index but they were highly collinear. Therefore, we opted to retain the peer effect item as one of the six comprising the neighborhood social problems index).

As shown in Supplementary Table S1, 49% of study participants were female and 54% were of Latino/a background. Slightly less than four in ten were the oldest child in the household. Approximately 15% of caregivers were of immigrant background. The primary caregiver was, on average, between 40 and 42 years of age. Approximately 31% of caregivers had no degree, 39 to 41% had completed a high school diploma or GED, and between 27 and 29% had completed a post-high school technical certification or college degree. Caregiver earnings averaged roughly $13,000 with a range from $0 to $66,352. The typical adolescent had 1.7 siblings and moved on average, 3.7 times during childhood. Households experienced, on average, 1.2 household stressors measured contemporaneously at the time of initiation of alcohol or tobacco use.

#### 2.3.4. Initiation of Alcohol and Tobacco Use During Adolescence

The outcomes of interest in our study are the initiation of alcohol or tobacco use during adolescence and the age of onset. For purposes of this study, adolescence is defined as the period from 10 through 18 years of age. These variables were operationalized by a question answered by caregivers about whether their child ever drank or used tobacco during childhood and if they did, the age when they first drank alcohol or smoked tobacco. To facilitate caregiver recall, event history or life calendars were used to obtain the data retrospectively. The collection of retrospective data from primary caregivers has been widely used in studies of child behaviors and outcomes across childhood (see for example, Panel Study of Income Dynamics, National Longitudinal Study of Youth, Fragile Families and Child Well-Being Study, and Three Cities Study). Previous studies have found that primary caregivers can recall and date major events and details about their children’s lives with a high degree of accuracy. Recall of the occurrence, location, and timing of significant life events, including substance use and exposure to violence, has been found to be sufficiently reliable for most epidemiological applications [120,121,122,123,124]. Moreover, errors in recall have not substantially affected parameter estimates of hazard models [121,125].

Nonetheless, we acknowledge the potential shortcomings of these retrospective, caregiver-reported data. First, they are subject to recall errors despite the use of event history calendars, landmark events, use of filters for complex behaviors, and single-focus items to reduce these errors [126,127]. Second, for outcomes such as adolescent substance use, these reports are based on caregiver perceptions. Although caregivers may have first-hand knowledge that serves as the basis of these perceptions, we note that their perceptions may not always be accurate because children may deliberately hide these experiences from them [128,129]. Third, they are subject to caregivers’ willingness to reveal extralegal behaviors (underage drinking or tobacco use) of their children to the interviewer. Although all three concerns likely created noise in our alcohol and tobacco use measures, we assume there is no systematic pattern in these errors related to neighborhood contexts.

Given these concerns about caregiver self-reports of alcohol and tobacco use, we compared these self-reports with past year prevalence rates and ages of initiation for adolescents in Colorado and the United States reported by the Substance Abuse and Mental Health Services Administration [13,130]. Since the 2023 National Survey on Drug Use and Health [1] detailed tables do not include national nor State of Colorado information for adolescent substance use initiation by race/ethnicity, we use the information available in the 2020 reports here. As shown in Table 2, the prevalence of alcohol use among adolescents in the study was higher (12.9% average; range 11.4 to 14.6%) than reports for Colorado and U.S. adolescents as a whole (10.2% and 9.1% respectively). Reported initiation of tobacco use was considerably higher for study participants (13.7% average; range from 12.3 to 17.3%) relative to those reported for Colorado (4.6%) and U.S. adolescents (2.2%). Additionally, the *Denver Child Study* conducted semi-structured follow-up interviews with a subset of 34 caregivers and separate interviews with their 45 young adult children in order to assess the validity of the parental reports from the retrospective survey relative to the young adult child self-reports. Using dyadic analysis of these data to examine childhood exposure to violence, Tate Woodson [131] found that 91% of the caregiver-young adult dyad reports of outcomes were congruent—not only did the dyad report the same events but shared similar perceptions about the neighborhood mechanisms associated with the events.

Slightly less than 13% of the study participants started using alcohol during adolescence with the average age of initiation at 16.1 years. About 14% began using tobacco as adolescents; the average age of initiation was 15.6 years. Prevalence rates for adolescents aged 12 to 17 residing in the State of Colorado were 10.2% for alcohol use with an average age of initiation of 13.2 years and 4.6% for tobacco use with an average age of initiation of 12.8 years [130]. Initial alcohol and tobacco use for both study and Colorado adolescents was also higher than for U.S. adolescents as a whole. Boys had higher rates of alcohol and tobacco use than girls, but girls initiated their use of both substances earlier than boys. Latino/a adolescents had higher rates of initiation than African American adolescents although the average age of initiation was about the same for alcohol use and about half a year younger for tobacco use by Latino/as. However, log-rank analyses of the survival curves for all gender and race/ethnic groups not shown here indicate no statistically significant group differences in the timing of either alcohol or tobacco use initiation during adolescence.

We also examined variations in alcohol and tobacco use by exposure to neighborhood violence. Adolescents residing in neighborhoods that had lower levels of neighborhood problems had significantly lower use of alcohol and tobacco (12.1%) and initiated their use at older ages (16.3 and 15.6 years, respectively, than adolescents residing in neighborhoods with higher levels (3 or more) of neighborhood problems (14.6% for alcohol use, 17.3% for tobacco use with age at onset of approximately 15.5 years). These data suggest that adolescents who lived in neighborhoods with above-average levels of exposure to violence had higher prevalence rates for using alcohol and tobacco than their peers overall (1.7 and 3.6 percentage points, respectively) and initiated that use 2 to 8 months earlier.

### 2.4. Analytical Approach

Data about sampled households, caregivers, adolescents, and their corresponding neighborhood environments were merged to create a pseudo-longitudinal database where the *child-year* became the unit of analysis. These data formed the basis for modeling how the neighborhood to which an adolescent’s family was quasi-randomly assigned by DHA subsequently affected their hazard of initiating alcohol or tobacco use during adolescence. Effectively, the “experimental treatment” that the DHA provides its households is a complex bundle of neighborhood attributes as noted above. We test the consequences of these various neighborhood exposures to violence by employing Cox proportional hazard (PH) regression models with clustered robust standard errors Since we are not following a specific cohort of adolescents (e.g., adolescents who moved into DHA at the same age and during the same year), we are not concerned about clustering at the neighborhood level. Adolescents in the *Denver Child Study* who happen to live in the same neighborhood experienced different values for neighborhood indicators because they resided in these neighborhoods during different years of their lives and different calendar years. However, we are concerned about clustering within families; hence the adjustment of our empirical models to account for siblings within the same family to estimate the hazards of initiating alcohol or tobacco use during adolescence. These data analyses were conducted using Stata (version 18, StataCorp, College Station, TX, USA).

#### Cox Proportional Hazard Specification

In our analyses of alcohol and tobacco use initiation, we only consider those instances that occurred after quasi-random assignment to a DHA public housing unit in order to preserve the value of the natural experiment for drawing causal inferences. Our analytical approach models the timing of initiation of alcohol or tobacco use at time t for an individual *ij* with covariate vector *χ* using a Cox proportional hazards (PH) model.

λ(t|*χ_ij_*) = λ_0_(t) exp(*β*_1_*χ*_1__*ij*_ + … +* β_n_χ_nij_*) = λ_0_(t) exp(*χ_ij_ β*)

where λ(t|*χ_ij_*) is the observed time of outcome (or the censoring time of age 18) for adolescent *ij* and λ_0_(t) is the baseline hazard. Global chi-square tests of proportionality indicate that the Cox PH models were the appropriate specifications to employ with these data:

Given the potential for multicollinearity across our neighborhood variables and other covariates in the model, we conducted several sensitivity tests which included predictors with variance inflation factors (VIFs) less than 3—values indicating either no or little collinearity—with the exception of our measures for neighborhood social vulnerability and household income which are retained for their theoretical relevance. Further, stratification was employed in order to test the extent to which relationships observed across study participants as a whole remained robust for females and males as well as Latino/a and African American race/ethnic groups. The theoretical and empirical rationale for exploring this heterogeneity is described in detail here [32,58,71,132].

## 3. Results

Standardized hazard ratios and robust standard errors for our models predicting the initiation of alcohol and tobacco use during adolescence are presented in Table 3 and Table 4. The interpretation for the hazard ratio is that a one standard deviation change in a given predictor (in this case neighborhood exposure to violence) is associated with an increase/decrease in the hazard of initiating alcohol or tobacco use during adolescence. Each table compares estimated parameters for adolescents as a whole as well as across gender and race/ethnic strata. In all models, all time-varying caregiver, household, and neighborhood conditions are measured contemporaneously when the adolescent first initiated use or at time-of-survey or at age 18 (whichever is younger) if the adolescent never used alcohol or tobacco during adolescence. Across all but one of the stratified models (African American tobacco use initiation), overall model performance was acceptable as demonstrated by the log-likelihood values and statistically significant chi-square tests.

### 3.1. Exposure to Neighborhood Violence and Adolescent Alcohol Use Initiation

Table 3 presents the results of our standardized Cox PH models for initiation of alcohol use during adolescence. Higher levels of neighborhood exposure to violence were associated with higher hazards of initiating adolescent alcohol use for study participants as a whole and for the adolescent male strata. A one standard deviation higher neighborhood social problems index score was associated with a 32% higher hazard of initiating alcohol use for adolescents as a whole; this effect was even stronger (38%) for adolescent males. However, exposure to neighborhood violence did not have a significant effect on alcohol initiation for female, Latino/a, or African American adolescent strata in the study.

### 3.2. Exposure to Neighborhood Violence and Adolescent Tobacco Use Initiation

Our neighborhood exposure to violence indicator provided consistently statistically significant predictors of the initiation of adolescent tobacco use across study participants as a whole and all of the strata except for female adolescents (see Table 4). Adolescents who resided in neighborhoods with one standard deviation-higher levels of social problems had a 36% greater hazard of initiating tobacco use during adolescence. The magnitude of these effects was higher for adolescent males (44%) but insignificant for females. However, the effects were slightly lower for Latino/a adolescents (32%) but notably higher for African American adolescents (50%).

### 3.3. Marginal Effects of Neighborhood Exposure to Violence on Predicted Hazards of Adolescent Alcohol and Tobacco Initiation

**Figure 1:**
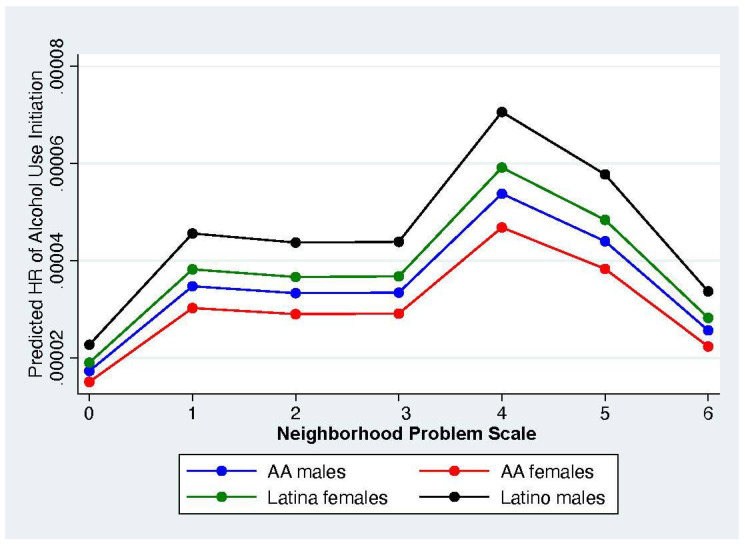
Marginal Effects at the Means for the Impact of Neighborhood Social Problems on the Predicted Hazard Ratio for Adolescent Alcohol Use Initiation by Gender and Race/Ethnicity.

**Figure 2:**
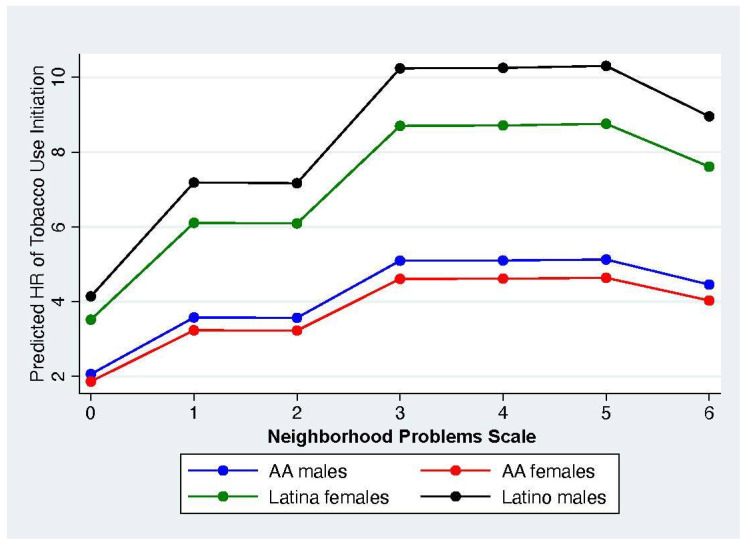
Marginal Effects at the Means for the Impact of Neighborhood Social Problems on the Predicted Hazard Ratio for Childhood Tobacco Use Initiation by Gender and Ethnicity.

The marginal effects and marginsplot features of Stata were then used to estimate the marginal effect at the means of the impact of exposure to neighborhood violence on the predicted hazard ratio of adolescent alcohol and tobacco use after controlling for other individual, caregiver, household, and other neighborhood contexts (see Figure 1 and Figure 2) (In order to make it easier to read the figures, we have not included the 95% confidence intervals (CIs) here. However, the marginal differences across neighborhood problems and across race/ethnic groups were not significant. Figures with the CIs are available from the first author upon request). These marginsplots depict the predicted hazard of initiating alcohol or tobacco use during adolescence for the average or typical adolescent residing in places with a given level of exposure to neighborhood social problems after controlling for the influences of the other covariates and provide us with a straightforward way to estimate potential threshold effects. These results show the marginal differences by gender and race/ethnic background.

As we can see in Figure 1, the predicted hazard ratio for adolescent alcohol use initiation is similar across all four groups. In order to differentiate between the four groups of adolescents, we have enlarged the figure by narrowing the scale on the Y axis. The predicted hazard ratio is essentially the same and relatively flat across the four groups with the exception of a slight increase in the hazard for adolescents residing in neighborhoods with four social problems before it decreases to levels that are nearly the same among neighborhoods with the highest and lowest number of neighborhood problems. For the typical adolescent, exposure to neighborhood violence has little influence on their initiation of alcohol use even in neighborhoods with the highest levels of social problems, nor does the pattern suggest any statistically significant nonlinearities or threshold effects for alcohol use initiation.

In contrast, changes in the hazard ratio for tobacco use initiation show some clear gender and race/ethnic differences relative to exposure to neighborhood violence for the typical adolescent resident (see Figure 2). The hazard ratio is relatively flat across neighborhoods where African American adolescents reside showing little variation in the risk of tobacco use regardless of level of exposure to violence. In contrast, the hazard ratios for tobacco initiation are highest for Latino men followed by Latina women. Additionally, the hazard ratio increases for Latino/a men and women and then essentially plateaus for neighborhoods with three, four, or five social problems before declining in neighborhoods with all six neighborhood social problems included in the index.

### 3.4. Robustness Checks

Given our use of the more localized neighborhood problems index instead of *Uniform Crime Report* measures or property and violent crime rates at the census tract or zip code level, we found in analyses not presented here consistent results indicating increased hazards of alcohol use initiation (range 53–70%) associated with increases in property crime rates for all adolescents as well as the female and Latino/a strata while neither crime measure was significant for adolescent males. Increased hazards of tobacco use initiation during adolescence (range 27–87%) were associated with increases in property crime rates for adolescents as a whole and male and female strata but were not significant for Latino/a or African American adolescents. Counterintuitive to expectations, increases in violent crime rates were found to decrease the risk of alcohol and tobacco use initiation for adolescents as a whole which may reflect enhanced surveillance by adults in areas with higher levels of violent crime against persons and therefore, reduced opportunities for underage use alcohol or tobacco. We also speculate that variations in these observed patterns of risk associated with neighborhood exposure to violence reflect differences in spatial scales since there would be significantly higher numbers of property and violent crimes reported as the neighborhood scale moves from the immediate areas surrounding the block of residence to the larger census tract. These analyses are available upon request from the first author.

## 4. Discussion

What are the effects of exposure to neighborhood violence on the prevalence and hazards of adolescent alcohol and tobacco use initiation? By age 18, 12.9% of adolescents in the study had initiated alcohol use and 13.7% initiated tobacco use. As hypothesized, prevalence rates for adolescent alcohol and tobacco use initiation were 1.7% and 3.6% higher, respectively, for adolescents residing in neighborhoods with higher exposure to violence compared to study adolescents as a whole. Moreover, prevalence rates were 2.6 and 5.2 percentage points higher for adolescents with higher exposure to violence relative to those residing in neighborhoods with lower levels of violence exposure. Additionally, the results support our hypothesis that greater exposure to neighborhood violence decreases the age of alcohol and tobacco use initiation. On average, adolescents in the study initiated alcohol use at age 16.1 years and tobacco use at age 15.6 years. However, adolescents initiated alcohol use at 15.5. years and tobacco use at 15.4 years, on average, roughly two to eight months younger, if they resided in neighborhoods with higher levels of violence—most likely as a coping response to increased levels of stress associated with the violence. After controlling for adolescent, caregiver, household, and other neighborhood confounders, exposure to neighborhood violence was associated with significantly higher hazards of alcohol and tobacco use initiation for study participants as a whole (32 and 36%, respectively).

Does the effect of neighborhood violence exposure on adolescent alcohol and substance use initiation vary significantly by gender and race/ethnicity after controlling for adolescent, caregiver, household, and other neighborhood contexts? Findings show that while males had higher prevalence rates for alcohol and tobacco use initiation than females—2.9 and 1.8 percentage points, respectively—females initiated use of both substances between 3 and 5 months earlier, on average, than males although gender differences were not statistically significant. After controlling for potential confounders, however, we found partial support for our hypothesis on gender differences. While exposure to higher levels of neighborhood violence had no significant effect on alcohol use initiation among adolescent females, the hazard of alcohol use among male adolescents was 39% higher. This is consistent with studies that suggest adolescent males are more likely to use alcohol for social enhancement (see discussion in [133]). Indeed, this is supported by qualitative data from male adolescents in the *Denver Child Study* who talked about using alcohol to ‘feel good, hang out with others, or because it was cool’. Prevalence rates for adolescent alcohol and tobacco use initiation in our study were comparable with other studies based on national survey samples (see [37,46,49]), the finding of higher prevalence among males relative to females is consistent with recent studies [40] although others suggest the gender gap is narrowing or nonsignificant for alcohol use [10,36]. The average age at the time of initiation of alcohol and tobacco use was also very similar to recent studies (e.g., [46,49]). We suspect that the higher prevalence among males also may reflect greater societal acceptance of drinking by ethnic minority male teens. Additionally, adolescent males may experience less adult surveillance of their activities or less adherence to such supervision and hence, enhanced opportunities to obtain alcohol and tobacco products in the neighborhood, especially for those living in neighborhoods with considerable gang and drug activity. White and colleagues [130] referred to the constellation of these conditions as socially toxic environments. Moreover, caregivers in the study noted how they carefully monitored the activities of their adolescents, and particularly their daughters minimizing the amount of unsupervised time outside or with friends in neighborhoods with higher exposure to violence. Also, some caregivers and female adolescents in the study noted they started smoking because of peer pressure or stress.

Additionally, the findings are mixed relative to our hypothesis about adolescent alcohol and tobacco use initiation among Latino/a and African American adolescents in our study. Consistent with prior studies [44,46,48], Latino/a prevalence rates of alcohol and tobacco use initiation were significantly higher than those for African American adolescents in our study. The age of initiation for both substances was slightly lower for Latino/a adolescents—age 16 for alcohol use initiation and 15.4 for tobacco use initiation. Although exposure to neighborhood violence did not predict the hazard of alcohol use for either African American or Latino adolescents in the study, it was a significant predictor of tobacco use initiation. After controlling for individual, caregiver, household, and other neighborhood confounders, Latino/a and African American adolescents had 32% and 50%, respectively, higher hazards of tobacco use initiation during adolescence. These findings are consistent with prior studies (e.g., [92,102,109]). that reported increased rates of adolescent substance use, particularly tobacco use with greater exposure to neighborhood or community violence. Further, compared to African American adolescents, Latino/a teens in Denver tend to reside in neighborhoods with greater marketing of drinking and smoking as well as greater access to alcohol and tobacco outlets.

Are there threshold effects between neighborhood violence exposure and adolescent alcohol and tobacco use initiation? We found some evidence of threshold effects on the influence of neighborhood exposure to violence on adolescent tobacco use initiation. Increasing hazards of tobacco use initiation plateaus between three and five neighborhood social problems, especially for Latino/a adolescents. We speculate that in neighborhood settings with multiple neighborhood social problems, there may be higher incidences of adolescents being exposed to or victimized by neighborhood violence. However, as the number of different social problems increases and clusters within a subset of neighborhoods, resident adolescents and their parents may become increasingly vigilant and hypervigilant, focusing on increasing surveillance of their surroundings as well as avoidance of the areas entirely. Indeed, exposure to heightened levels of violence was the neighborhood mechanism most frequently identified by caregivers in our study affecting their adolescent children. Their primary response was to keep their adolescents away from dangerous neighbors and areas in the neighborhood, not let them walk by themselves or without a caregiver, or to literally participate in activities located outside of the neighborhoods where they resided. Once an area is perceived as dangerous, any additional problems may cement fear and avoidance but produce diminishing returns to any additional impact on the hazards of tobacco use initiation since there are fewer opportunities to target adolescents in those areas. Nonetheless, these heightened perceptions of danger or feeling unsafe trigger psychological reactions such as stress, which in turn, may lead to initiating the use of tobacco (by all subgroups of adolescents) as a way of coping. While our findings about adolescent tobacco use initiation are consistent with prior studies [98,102,103,104,109], our study provides new information about possible threshold effects.

Other potential pathways that may foster adolescent alcohol and tobacco use initiation include adolescents’ attitudes, values, behaviors, and expectations about alcohol and tobacco use shaped by weaker collective norms proscribing alcohol and tobacco or unenforced by the community or local culture [26,27,56,66,80,87,92,98]. These may be further reinforced by the presence of neighborhood adult role models who drink or smoke that adolescents may choose to emulate. Adolescents may also reside in neighborhoods where residents are unable to exert their collective efficacy in resolving neighborhood problems. The lack of collective efficacy may create an environment where not only neighborhood violence but also adolescent alcohol and tobacco use go unchallenged in public spaces. Finally, areas with higher levels of neighborhood violence or other forms of disadvantage may also be targeted with aggressive marketing campaigns glamorizing substance use and the concentration of alcohol and tobacco outlets—both facilitating adolescent access to those substances [60,81,105].

## 5. Conclusions, Caveats, and Future Directions

Data from a natural experiment involving low-income ethnic minority adolescents were used to assess potential causal neighborhood and threshold effects on adolescent substance initiation. Our findings expand the literature examining how exposure to neighborhood violence influences alcohol and tobacco use initiation during adolescence and how these effects vary for Latino/a and African American adolescents and by gender. We employed Cox PH models to estimate parameters and found that contemporaneous exposure to neighborhood violence strongly and robustly predicted the initiation of alcohol or tobacco use during adolescence. Further, we found suggestive evidence of threshold effects with diminishing returns for neighborhood violence exposure on adolescent tobacco use initiation.

Consistent with prior studies that examine a high degree of heterogeneity in neighborhood effects by gender and race/ethnicity, we also found such heterogeneity in the effects of exposure to neighborhood violence on adolescent alcohol and tobacco use initiation [32,65,67,83]. After controlling for other confounders, exposure to neighborhood violence proved to have a stronger influence on alcohol use initiation for adolescent males and tobacco use initiation for Latino/a and African American adolescents. Thus, our study contributes to the growing body of evidence on effect heterogeneity and suggests that both the substance abuse literature as well as neighborhood effects literature need to assess for whom, when, and where these effects matter [32].

Although we believe our use of quasi-experimental data advances the literature on adolescent substance abuse initiation, we recognize several limitations in the study. As previously noted, our measures of adolescent alcohol and tobacco use initiation are derived from retrospective caregiver reports which are subject to recall and reporting errors. However, if present, such errors would effectively render any neighborhood effect findings insignificant. The data also do not capture information about adolescents’ subsequent alcohol and tobacco use so we were unable to examine the extent to which such use continued after initiation. Nor do the data provide us with detailed information about motivations which could further disentangle the mechanisms behind the gender and ethnic differences that were found. Also, because of data and sample size limitations, we did not examine the various pathways through which adolescent substance use initiation might mitigate other adolescent outcomes. This in part reflects the lack of in-depth information for all of these potentially intervening circumstances. Finally, we recognize that our findings from this case study in Denver are not generalizable to all Latino/a and African American adolescents nor for ethnic minority adolescents from low-income families.

Nonetheless, our findings underscore the importance of neighborhood as a developmental context influencing adolescent alcohol and tobacco use initiation among low-income Latino/a and African American adolescents and likely, ethnic minority adolescents in general. Our findings recognize that while adolescent, caregiver, and household contexts play an important role in alcohol and tobacco use initiation among teens, neighborhood contexts, and particularly exposure to neighborhood violence, can heighten risks for adolescents. Further, the early onset of alcohol and tobacco use can trigger serious yet preventable behavioral and physical health outcomes that can affect health and well-being throughout the lifespan [13]. Therefore, practitioners and policymakers need to address the role that neighborhood contexts play not only in adolescent alcohol and tobacco use initiation but also in shaping their future opportunities and life chances.

One of the key challenges facing communities is providing all adolescents the opportunity to live in healthy and safe neighborhoods and homes offering supportive environments throughout childhood and into adulthood [13,46,50,134]. Designing opportunity-rich housing and neighborhoods is crucial to ensuring that disadvantaged teens gain better access to places that enhance their opportunities. Additionally, community-based initiatives that reduce access to alcohol and tobacco outlets as well as aim to decrease neighborhood social problems and corresponding violence would go far to reduce Latino/a and African American adolescent alcohol and tobacco use initiation. For instance, programs like *Communities that Care* and *Communities Mobilizing for Change on Alcohol* engage in collective efforts to reduce advertising and limit business hours for alcohol sales, reduce the density of alcohol and tobacco outlets, raise the minimum legal age for the purchase and consumption of alcohol and tobacco, and enforce laws targeting underage drinking and smoking [13]. Community-led, evidence-based programs, such as *Rise Above Colorado* [134], support efforts promoting positive youth development and fostering positive community norms as prevention and early intervention strategies. Facilitated by the teens themselves through their Teen Action Councils, *Rise Above Colorado* offers adolescents life skills training, education, and positive community norming activities aimed at deterring substance misuse. Community members participating in the program also engage in community awareness and positive community norming activities as well as enhancing neighborhood safety through the formation of neighborhood watch groups [134]. Critical to the reduction of violence is the creation of safe public spaces with prosocial activities that enhance neighborhood safety. Neighborhood youth clubs, sports teams, community centers, and parks may serve as powerful deterrents to substance use initiation among adolescents [50]. Community- or school-based instruction, starting in elementary school, could focus on prevention as well as the effects of adolescent substance abuse on health over the life course. Evidence-based programs such as *Life Skills Training*, *Keepin’ It REAL*, *Project Toward No Drug Abuse*, *Raising Healthy Children*, and *Health Rocks* among others [13,46,134,135,136,137] hold promise as community-level interventions. However, additional work is needed to develop programming and activities that facilitate these efforts in ethnic minority communities.

## Figures and Tables

**Table 1:** Descriptive Characteristics of Neighborhoods for Study Populations and City and County of Denver.

	Alcohol Use (N = 1060)				Cigarette Use (N = 1093)				Denver Average During Adolescence			
	Mean	*SD*	Min	Max	Mean	*SD*	Min	Max	Mean	*SD*	Min	Max
***Neighborhood Contexts (all continuous variables measured at time of initiation or age 18 if no use)***												
***Exposure to Neighborhood Violence***												
Social problems index (range 0–6)	1.89	1.78	0.00	6.00	1.89	1.78	0.00	6.00	NA	NA	NA	NA
***Neighborhood Controls***												
Social vulnerability score (range 0–400)	118.93	52.26	23.19	288.97	119.16	53.63	19.86	288.97	96.73	42.01	16.26	275.30
Percent African American residents	14.26	17.27	0.07	99.51	14.97	17.91	0.07	99.51	11.54	16.66	0.13	75.26
Percent Latino/a residents	55.39	22.67	0.09	96.10	54.48	22.55	0.09	96.10	28.87	24.63	2.12	84.02
Occupational prestige score (range 29–62)	37.44	3.45	31.36	48.34	37.42	3.45	30.15	49.88	41.01	4.37	32.33	50.14
Percent of housing built before 1940	23.67	19.06	0.00	97.86	23.83	19.65	0.00	97.86	25.06	25.67	0.00	84.57

Source: Compiled by authors using *Denver Child Study* data. Data for the social problem index were not available for the City and County of Denver.

**Table 2:** Prevalence and Age at Initiation of Adolescent Alcohol and Cigarette Use * for *Denver Child Study* Participants, Colorado, and U.S. Adolescents **.

	Mean	2019 Average Colorado Adolescents	2019 Average U.S. Adolescents
**Alcohol Use**			
***Ever drank alcohol (%)***			
All study participants (N = 1062)	12.9	10.2	9.1
Males (N = 537)	14.3		8.3
Females (N = 525)	11.4		9.9
Latino/as (N = 569)	14.2		9.4
African Americans (N = 493)	11.4		6.3
Social Problem Index LT 3 (N = 741)	12.1		NA
Social Problem Index GTE 3 (N = 321)	14.6		NA
***Age in years at time of initiation of alcohol use***			
All study participants (N = 137)	16.1	13.2	NA
Males (N = 77)	16.3		NA
Females (N = 60)	15.8		NA
Latino/as (N = 81)	16.0		NA
African Americans (N = 56)	16.1		NA
Social Problem Index LT 3 (N = 90)	16.3		NA
Social Problem Index GTE 3 (N = 47)	15.5		NA
**Cigarette Use**			
***Ever smoked cigarettes (%)***			
All study participants (N = 1093)	13.7	4.6	2.2
Males (N = 555)	14.6		2.2
Females (N = 538)	12.8		2.2
Latino/as (N = 587)	15.0		2.2
African Americans (N = 506)	12.3		0.8
Social Problem Index LT 3 (N = 758)	12.1		NA
Social Problem Index GTE 3 (N = 335)	17.3		NA
***Age in years at time of initiation of cigarette use***			
All study participants (N = 150)	15.6	12.8	NA
Males (N = 81)	15.7		NA
Females (N = 69)	15.4		NA
Latino/as (N = 88)	15.4		NA
African Americans (N = 62)	15.8		NA
Social Problem Index LT 3 (N = 92)	15.6		NA
Social Problem Index GTE 3 (N = 58)	15.4		NA

Notes: * Prevalence and age of onset were based on caregiver retrospective reports of these behaviors for children aged 10 and older at the time of the *Denver Child Study* survey. Please note that these were similar to self-reported rates of 12% (drinking); 12% (smoking); and 7% (marijuana use) from the Project on Human Development in Chicago Neighborhoods (see [96]). ** Prevalence rates for the U.S. and Colorado derived from Substance Abuse and Mental Health Services Administration (2020a) [2]. *Behavioral Health Barometer, United States, Volume 6: Indicators as measured through the 2019 National Survey on Drug Use and Health and the National Survey of Substance Abuse Treatment Services*. Publication No. PEP20-07-02-001. Rockville, MD: Substance Abuse and Mental Health Services Administration; Substance Abuse and Mental Health Services Administration (2020b) [130]. *Behavioral Health Barometer, Colorado, Volume 6: Indicators as measured through the 2019 National Survey on Drug Use and Health and the National Survey of Substance Abuse Treatment Services*. HHS Publication No. SMA-20-Baro-19-CO. Substance Abuse and Mental Health Services Administration, Rockville, MD, USA. Information for age at time of initiation is calculated differently in the SAMHSA reports so this information is not reported here.

**Table 3:** Standardized Cox Proportional Hazards Models Predicting Initiation of Alcohol Use During Adolescence by Gender and Ethnicity.

	Full Sample		Male		Female		Latino		African American	
	HR	SE	HR	SE	HR	SE	HR	SE	HR	SE
**Predictor Measures (All continuous variables reflect standardized values measured at time of initiation)**										
***Exposure to Neighborhood Violence***										
Social problems index score (range 0–6)	1.32 *	(0.16)	1.38 **	(0.16)	1.32	(0.27)	1.33	(0.20)	1.44	(0.27)
***Neighborhood Controls***										
Social vulnerability index score (range 0–400)	1.10	(0.10)	1.21	(0.16)	1.12	(0.15)	1.09	(0.15)	1.18	(0.22)
Percentage African American residents	0.58 **	(0.09)	0.35 ***	(0.08)	0.86	(0.17)	0.57	(0.20)	0.56 *	(0.13)
Percentage Latino residents	0.64 **	(0.11)	0.50 ***	(0.10)	0.82	(0.20)	0.62 **	(0.11)	0.54	(0.21)
Occupational prestige score (range 29–62)	0.70 *	(0.12)	0.56 **	(0.11)	0.93	(0.31)	0.58 *	(0.13)	0.68	(0.25)
Percentage of housing units built before 1940	1.10	(0.14)	1.09	(0.14)	1.15	(0.26)	1.12	(0.14)	1.15	(0.31)
***Adolescent Controls***										
Gender and ethnicity (omitted = African American male)										
Latina female	1.04	(0.31)								
Latino male	1.36	(0.34)								
African American female	0.87	(0.26)								
African American (omitted = no)			0.93	(0.22)	0.66	(0.26)				
Female (omitted = no)							0.76	(0.17)	1.02	(0.31)
First born in family (omitted = no)	0.68 *	(0.11)	0.56 **	(0.12)	0.74	(0.21)	0.79	(0.16)	0.49 *	(0.14)
***Caregiver and Household Controls***										
Number of siblings in household	0.63 ***	(0.07)	0.63 **	(0.09)	0.55 ***	(0.09)	0.80 *	(0.09)	0.40 ***	(0.08)
Caregiver reported depressive symptomatology (omitted = no)	1.45	(0.37)	1.76	(0.51)	1.36	(0.49)	2.11 *	(0.68)	0.89	(0.34)
Caregiver age	0.28 ***	(0.05)	0.24 ***	(0.05)	0.28 ***	(0.07)	0.27 ***	(0.06)	0.23 *** ^a^	(0.07) ^a^
Caregiver immigrant status (omitted = no)	0.53	(0.25)	0.89	(0.41)	0.21	(0.22)	0.44	(0.24)		
Caregiver educational attainment (omitted = no degree)										
H.S. diploma	1.58	(0.37)	1.78	(0.53)	1.24	(0.42)	1.06	(0.30)	3.35	(2.21)
Post-H.S. technical certificate or college degree	1.56	(0.46)	1.85	(0.60)	1.21	(0.57)	1.05	(0.40)	3.36	(2.17)
Natural log of caregiver earnings (in dollars)	1.05	(0.11)	0.87	(0.10)	1.46	(0.32)	1.28	(0.18)	0.91	(0.18)
Household stressor scale (0–5)	0.97	(0.11)	0.93	(0.12)	1.06	(0.19)	1.01	(0.13)	1.06	(0.19)
Number of moves from birth to onset	0.94	(0.08)	0.84	(0.09)	1.07	(0.12)	0.93	(0.12)	0.95	(0.12)
Number of observations	1062.00		537		525		569		493	
Number of clusters	524.00		377		347		278		248	
Number of failures	137.00		77		60		81		56	
Time at risk	16,181.00		8166		8015		8655		7526	
Log-Likelihood	−787.97		−375.62		−307.40		−409.25		−273.35	
Chi-square	137.74 ***		138.26 ***		67.64 ***		89.45 ***		71.99 ***	

Notes: HR = hazard ratios (exponentiated coefficients); robust standard errors in parentheses. ^a^ Immigrant status was perfectly predictive in the African American model and thus was excluded. * *p* < 0.05; ** *p* < 0.01; *** *p* < 0.001.

**Table 4:** Standardized Cox Proportional Hazards Models Predicting Initiation of Cigarette Use During Adolescence by Gender and Ethnicity.

	Full Sample		Male		Female		Latino		African American	
	HR	SE	HR	SE	HR	SE	HR	SE	HR	SE
**Predictor Measures (All continuous variables reflect standardized values measured at time of initiation)**										
**Exposure to Neighborhood Violence**										
Social problems index score (range 0–6)	1.36 **	(0.13)	1.44 **	(0.17)	1.30	(0.20)	1.32 *	(0.17)	1.50 *	(0.26)
***Neighborhood Controls***										
Social vulnerability index score (range 0–400)	1.29 **	(0.12)	1.30 *	(0.15)	1.31 *	(0.17)	1.59 **	(0.28)	1.30	(0.23)
Percentage African American residents	1.07	(0.15)	0.95	(0.18)	1.26	(0.22)	0.47	(0.23)	1.48	(0.32)
Percentage Latino residents	0.96	(0.18)	0.90	(0.23)	1.04	(0.28)	0.72	(0.13)	1.62	(0.55)
Occupational prestige score (range 29–62)	1.19	(0.21)	1.11	(0.26)	1.29	(0.33)	0.93	(0.20)	1.79	(0.58)
Percentage of housing units built before 1940	0.86	(0.11)	0.77	(0.11)	0.96	(0.16)	1.02	(0.14)	0.72	(0.14)
***Adolescent Controls***										
Gender and ethnicity (omitted = African American male)										
Latina female	1.59	(0.51)								
Latino male	1.88 *	(0.55)								
African American female	0.93	(0.25)								
African American (omitted = no)			0.62	(0.18)	0.45 *	(0.16)				
Female (omitted = no)							0.81	(0.19)	0.88	(0.24)
First born in family (omitted = no)	0.80	(0.13)	0.88	(0.21)	0.72	(0.19)	0.76	(0.18)	0.84	(0.21)
***Caregiver and Household Characteristics***										
Number of siblings in household	0.67 ***	(0.07)	0.57 ***	(0.09)	0.71 **	(0.09)	0.73 *	(0.10)	0.68 *	(0.12)
Caregiver reported depressive symptomatology (omitted = no)	1.46	(0.36)	1.12	(0.38)	2.06 *	(0.64)	2.49 **	(0.7)	0.87	(0.35)
Caregiver age	0.63 **	(0.09)	0.53 **	(0.10)	0.73	(0.15)	0.54 **	(0.10)	0.81	(0.17)
Caregiver immigrant status (omitted = no)	0.62	(0.24)	1.77	(0.66)	0.14	(0.15)	0.58	(0.26)	1.12	(0.90)
Caregiver educational attainment (omitted = no degree)										
H.S. diploma	1.14	(0.26)	1.10	(0.31)	1.09	(0.35)	0.85	(0.24)	1.49	(0.65)
Post-H.S. technical certificate or college degree	0.83	(0.23)	0.73	(0.28)	0.77	(0.29)	0.86	(0.28)	0.75	(0.40)
Natural log of caregiver earnings (in dollars)	1.05	(0.11)	1.09	(0.14)	1.05	(0.16)	1.09	(0.17)	1.03	(0.17)
Household stressor scale (0–5)	1.11	(0.12)	1.21	(0.17)	1.05	(0.18)	1.15	(0.19)	1.05	(0.16)
Number of moves from birth to onset	0.98	(0.09)	1.00	(0.14)	0.97	(0.11)	0.99	(0.13)	1.00	(0.14)
Number of observations	1093		555		538		587		506	
Number of clusters	533		386		356		285		250	
Number of failures	150		81		69		88		62	
Time at risk	16,625		8424		8201		8897		7728	
Log-Likelihood	−923.93		−436.95		−374.44		−474.11		−334.02	
Chi-square	70.29 ***		59.00 ***		39.55 **		85.82 ***		20.21	

Notes: HR = hazard ratios (exponentiated coefficients); robust standard errors in parentheses. * *p* < 0.05; ** *p* < 0.01; *** *p* < 0.001.

## Data Availability

The datasets presented in this article are not readily available because they are under a Data Confidentiality Agreement with the Denver Housing Authority. Requests for the data analyses supporting the research in this study should be directed to first author.

## Supplementary Materials

The following supporting information can be downloaded at: https://www.mdpi.com/article/10.3390/healthcare13020194/s1.
