# Supplementary material for: Neighborhood Violence Exposure and Alcohol and Tobacco Use Initiation Among Ethnic Minority Adolescents
Source: Healthcare (Basel). 2025 Jan 19;13(2):194. doi: 10.3390/healthcare13020194 (PMC11764535; doi:10.3390/healthcare13020194)
Supplement: Supplementary file 1 [file healthcare-13-00194-s001.zip › healthcare-3300923-supplementary.pdf]

Supplementary Table S1. Descriptive Characteristics of Youth, Caregivers and Households for Analysis Samples

	Alcohol Use (N=1,062)				Tobacco Use (N=1,093)			
	**Mean**	***SD***	**Min**	**Max**	**Mean**	***SD***	**Min**	**Max**
**Predictor Measures**								
Youth Characteristics								
Gender and race/ethnicity of youth								
Latina female	0.26	0.44	0.00	1.00	0.26	0.44	0.00	1.00
Latino male	0.28	0.45	0.00	1.00	0.28	0.45	0.00	1.00
African American female	0.23	0.42	0.00	1.00	0.23	0.42	0.00	1.00
African American male	0.23	0.42	0.00	1.00	0.23	0.42	0.00	1.00
First born in family (omitted=no)	0.38	0.48	0.00	1.00	0.38	0.48	0.00	1.00
Caregiver and Household Characteristics (all continuous variables measured at time of initiation)								
Number of siblings in household	1.66	1.35	0.00	7.00	1.66	1.34	0.00	6.00
Caregiver reported depressive symptomatology	0.23	0.42	0.00	1.00	0.24	0.43	0.00	1.00
Caregiver age	41.88	8.49	25.47	78.94	40.36	7.77	25.47	78.94
Caregiver immigrant status (omitted=no)	0.15	0.36	0.00	1.00	0.15	0.36	0.00	1.00
Caregiver educational attainment (omitted=no degree)								
H.S. diploma	0.40	0.49	0.00	1.00	0.42	0.49	0.00	1.00
Post-H.S. technical certificate or college degree	0.29	0.45	0.00	1.00	0.27	0.44	0.00	1.00
Caregiver earnings (in U.S. dollars)	13029.01	12675.66	0.00	66352.00	12943.45	12553.79	1.00	66352.00
Natural log of caregiver earnings	6.52	4.58	0.00	11.10	6.54	4.56	0.00	11.10
Household stressor scale (range 0-5)	1.22	1.20	0.00	5.00	1.23	1.19	0.00	5.00
Total number of moves from birth to initiation	3.66	2.53	0.00	16.00	3.68	2.53	0.00	16.00

Source: Compiled by authors using *Denver Child Study* data.
